# 3*D* Information-Theoretic Analysis of the Simplest Hydrogen Abstraction Reaction

**DOI:** 10.1021/acs.jpca.3c01957

**Published:** 2023-07-21

**Authors:** Rodolfo O. Esquivel, Moyocoyani Molina-Espíritu, Sheila López-Rosa

**Affiliations:** † Departamento de Química, Universidad Autónoma Metropolitana, 09340 México D.F., México; ‡ Instituto Carlos I de Física Teórica y Computacional, Universidad de Sevilla, 41012 Sevilla, Spain; § Departamento de Física Aplicada II, Universidad de Sevilla, 41012 Sevilla, Spain; ⊥ Independent researcher, Querétaro 76125, Mexico

## Abstract

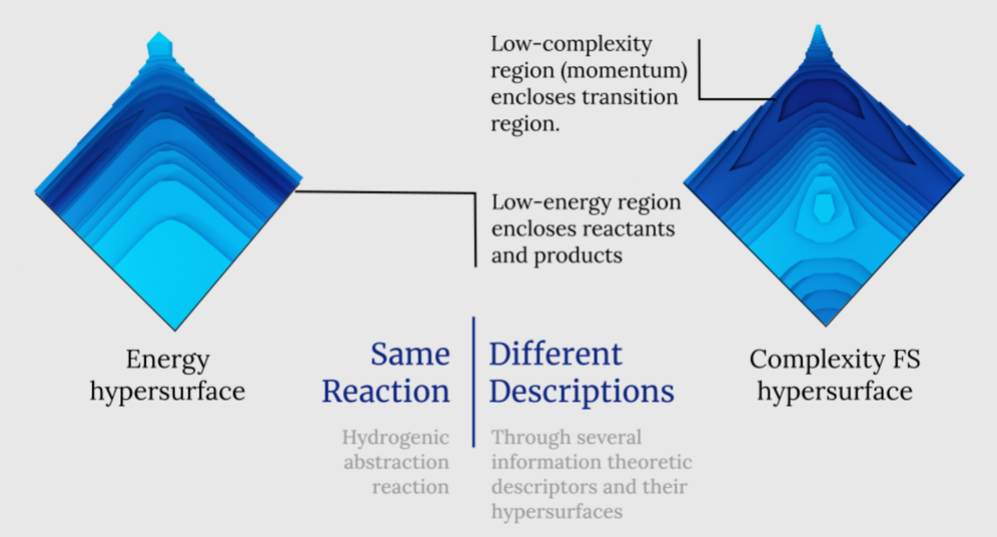

We investigate the course of an elementary chemical reaction from the perspective of information theory in 3*D* space through the hypersurface of several information-theoretic (*IT*) functionals such as disequilibrium (*D*), Shannon entropy (*S*), Fisher information (*I*), and the complexity measures of Fisher–Shannon (*FS*) and López–Mancini–Calbet (*LMC*). The probe for the study is the hydrogenic identity abstraction reaction. In order to perform the analysis, the reactivity pattern of the reaction is examined by use of the aforementioned functionals of the single-particle density, which is analyzed in position (*r*) and momentum (*p*) spaces. The 3*D* analyses revealed interesting reactivity patterns in the neighborhood of the intrinsic reaction coordinate (IRC) path, which allow to interpret the reaction mechanism for this reaction in a novel manner. In addition, the chemically interesting regions that have been characterized through the information functionals and their complexity measures are depicted and analyzed in the framework of the three-dimensional structure of the information-theoretical data of a chemical reaction, that is, the reactant/product (R/P) complexes, the transition state (TS), and the ones that are only revealed through *IT* measures such as the bond-cleavage energy region (BCER), the bond-breaking/forming (B-B/F) region, and the spin-coupling (SC) process. Furthermore, focus has been placed on the diagonal part of the hypersurface of the *IT* functionals, aside from the IRC path itself, with the purpose of analyzing the dissociation process of the triatomic transition-state complex that has revealed other interesting features of the bond-breaking (B-B) process. In other respects, it is shown throughout the combined analyses of the 3*D* structure of the *IT* functionals in conjugated spaces that the chemically significant regions occurring at the onset of the TS are completely characterized by information-theoretic aspects of localizability (S), uniformity (D), and disorder. Further, novel regions of low complexity seem to indicate new boundaries for chemically stable complex molecules. Finally, the study reveals that the chemical reaction occurs at low-complexity regions, where the concurrent phenomena take place: bond-breaking/forming (B-B/F), bond-cleavage energy reservoirs (BCER), spin-coupling (SC), and transition state (TS).

## Introduction

Understanding the stereochemical features of the passage from reactants to products has been the subject of great interest in Chemistry during the last decades; much work has been devoted to the analysis of potential-energy hypersurfaces at different levels of theory.^1^ Within the widespread ambit of these investigations, much research has been devoted on analyzing the stationary points of the energy surface. Within the Born–Oppenheimer approximation, minima on the potential-energy surface have been associated with molecular structures in dynamical equilibria and with saddle points to the transition states (TSs). Since the concept of the transition-state theory was formulated,^2,3^ much work has been dedicated to modeling and characterizing the transition state (TS). Due to this concept, it is possible to understand the chemical reaction barrier and hence to gain insight into the chemical reactivity. Quantum chemistry has circumvented the inherent complexity of the computational problem by assigning rigorous topological features of critical points on a potential-energy surface and thus associating them to chemical complexes in equilibrium and hence to the TSs. Therefore, the approach fully characterizes the energy minima and saddle points by assigning them to the first derivatives of the energy through the gradient (over the spatial configuration of the nuclei) and the second ones through the Hessian, respectively.^4−14^ In spite of the fact that critical points of the potential-energy hypersurface are useful for studying the chemical course of the reaction,^4^ these are only mathematical concepts and hence their chemical meanings are dubious.^15^ Understanding the TS is an essential goal of chemical reactivity theories, in order to achieve a deeper knowledge of chemical reactions in relation to their kinetics and its dynamics as well. In this context, various studies have employed density descriptors to analyze the course of chemical reactions,^2,16^ amongst which the reaction force analyses^17−19^ on the potential energy of reactions are worth mentioning to characterize the structural changes of electron densities during the course of chemical reactions. Moreover, Shi and Boyd^20^ undertook a systematic study of model *S*_*N*_2 reactions to study the features of the TS charge distribution by invoking the Hammond–Leffler postulate. Further, the topological properties of the Laplacian of the density were analyzed by Bader et al.^21^ who developed a theory of reactivity associating charge concentration/depletion with chemical regions of interest during the course of a chemical reaction. Moreover, Balakrishnan et al.^22^ showed that information-theoretic entropies increase to a maximum in a dynamical study when studying the time evolution of a bimolecular exchange reaction. Within the context of Information Theory, Shannon entropy studies revealed geometrical aspects of the density that are not present in the energy profile of elementary *S*_*N*_2 reactions.^23^ Moreover, Knoerr et al.^24^ found correlations between charge density aspects of the *S*_*N*_2 reaction and the energy-related measures of Shaik et al.^25^ Later, Tachibana^26^ identified the chemical bond-forming of model reactions through the kinetic energy density to identify several stages of the reactions along the IRC. Also, long-range dipole moments have been useful for ion–molecule reactions to control their reaction path with laser fields.^27^ Further, double proton transfer reactions were employed to show that changes in polarizations served to locate the transition-state structures.^28^ In spite of the aforementioned efforts for characterizing the transition-state region, it is difficult to achieve a complete description of the reaction mechanism of the elementary reaction. This study is focused to pursue such a goal through Information-Theory concepts.

Over the past decades, Information Theory has provided very useful mathematical and conceptual tools to study quantum systems, such as atoms, molecules, biological systems, mesoscopic materials, etc., in several correlated fields of Physics, Chemistry, and Biology. In line with the above, multidisciplinary research projects are being developed at different conceptual levels of theory: On the classical side through several uncertainty measures such as the Shannon entropy, the Fisher information, statistical complexity, etc., and on the quantum perspective through several entanglement measures and the essential concepts of locality and separability, which are being applied on a variety of quantum systems and processes.^29,30^

Indeed, atomic and molecular systems can be fully characterized by using a variety of uncertainty measures that depend on the electron density distribution, thus complementing the common energy representation, both obtained with the Schroedinger equation and its corresponding wavefunction or by employing density functional methods. The essentials of these complementary view depart on the description of the properties of physical and chemical systems based on several uncertainty measures such as the entropic character of the electron density,^31,32^ which measures the spreading of the distribution in turn associated with several chemical features of the densities (position and momentum) such as its localizability or depletion. These measures of uncertainty can also serve as indicators of disorder, uniformity, equiprobability, fluctuation, similarity, narrowness, complexity, etc., that are the basic ingredients of which many physicochemical properties depend on and also for describing numerous quantum phenomena of physical and chemical interest. Extensive research on these uncertainty measures has shown that information-theoretical functionals provide a useful description of atoms and molecules and their associated processes. For instance, the chemical description of the course of selected elementary chemical reactions revealed bond-forming and bond-breaking regions that are absent from the energy profile.^30^ This was achieved through localized/delocalized features of electron distributions shown by Shannon entropies, also with the uniformity/equiprobability features revealed by the disequilibrium measure and with the oscillatory/disorder features of the electron distributions shown by Fisher.^33,34^ Liu et al.^35−37^ quantified several descriptors of density functional theory (DFT), such as electrophilicity and nucleophilicity, among others, by means of the information conservation principle. Further, information theoretic (*IT*) measures were used to analyze molecular communication on molecules of biological systems.^38^ Despite the fact that Shannon entropy remains the most useful descriptor in *IT*, there is another uncertainty descriptor which is important in its own right and has gained wide attention in the recent years. Fisher information represents the kernel operator of fundamental motion equations through the minimum Fisher information principle^31,39^ in Physics, such as the nonrelativistic quantum-mechanical equations, the time-independent Kohn–Sham equations, and the time-dependent Euler equation.^40,41^ On the other hand, Fisher information, as an uncertainty descriptor, has been associated with local changes of the distributions, such as disorder, narrowness, fluctuation, and irregularity. For instance, the steric effect in ethane has been analyzed by means of the “narrowness” of the electron densities in position and momentum spaces.^42^ Moreover, tumor growth dynamics has been studied through Fisher information by applying extreme physical information analysis.^43^ Recently, multidimensional *IT* space based upon Shannon entropy, Fisher, and disequilibrium has been defined in order to characterize and classify atomic and molecular systems.^44^*IT* concepts such as spreading, equiprobability, and disorder, along with the shape complexity products (*LMC* and *FS*), reveal the particular aspects of a large and diverse number of many-electron systems, from atomic systems, compact molecules to very complex systems of biological interest, such as amino acids and pharmacological systems. This topological map might be used as an alternative framework of the chemical space,^45,46^ a multidimensional descriptor space based on the use of a great number of physicochemical properties as descriptors. On the other hand, information entropy has also been employed on chemical informatics by digitalizing chemical reactions, chemical synthesis, crystal engineering, and structural chemistry, among others.^47^

The aim of the study is at the core of the *IT* analysis of a 3*D* surface in which the pathway of a chemical reaction occurs so as to examine the “terrain” in which the reactants convert into products in their passage through the TS state. This is performed in intrinsic reaction coordinates (IRC), which are mass-weighted Cartesian coordinates that trace the transition state of a reaction toward its reactants (reverse direction) and products (forward direction). Therefore, the IRC represents a minimum energy reaction pathway (MERP) that resembles a chemical road crossing through a 3*D* horse saddle that forms an energy hypersurface. We have built such a 3*D*-saddle surface by extending the internal coordinates of the three hydrogenic complex *H*_*a*_ ··· *H*_*b*_ ··· *H*_*c*_ in all directions by use of an arbitrary equidistant grid running from 0.5*a.u*. ≤ *R*_12_ ≤ 3.35a*.u*. vs. 0.5*a.u*. ≤ *R*_13_ ≤ 3.35*a.u*. in steps of 0.05*a.u*. far beyond the IRC. Once the grid is formed, a 3D information-theoretical analysis of the hydrogenic abstraction reaction *H*_*a*_^•^ + *H*_2_ ⇋ *H*_2_ + *H*_*b*_^•^ is performed by use of information-theoretical measures and the dyadic produtcs of statistical complexity (*LMC* and *FS*). Focus will be set on the recognition of 3*D*-patterns of localizability, disorder and uniformity through the hypersurfaces of *S*, *I* and *D*, respectively.

The organization of the paper is as follows: (i) first, we define the *IT* functionals and its complexity joint measures, (ii) second, we identify the concurrent phenomena occurring at the course of the IRC path of the reaction through the *IT* functionals and its complexity dyadic measures, (iii) third, we calculate the 3*D* surfaces of the *IT* components along with the Fisher–Shannon and *LMC* complexities in position (*r*) as well as in momentum (*p*) spaces. The diagonal path of the IT-functionals hypersurfaces will be examined at the light of the equidistant dissociation of the three-atomic complex molecule at the TS *H*_*a*_ ··· *H*_*b*_ ··· *H*_*c*_. Finally, (iv) we present the discussion and conclusions from our results.

## Information-Theoretic Measures

The probability distributions for a molecule, i.e., the total electron density ρ(*r⃗*) in position space and the momentum-space density γ(*p⃗*) in the momentum ones, are given by the sum of the occupied electronic orbitals in the independent-particle approximation: the molecular position-space orbitals ψ_*i*_(*r⃗*) and the molecular momentals (momentum-space orbitals) ϕ_*i*_(*p⃗*), respectively. In turn, the momentals can be obtained by three-dimensional Fourier transformation of the corresponding orbitals (and conversely)
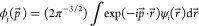
1it is worthy to mention that atomic units (a.u.) are employed to define eq 1 and this will be used in what follows. Standard procedures for the Fourier transformation of position-space orbitals generated by ab initio methods have been described.^48^ The orbitals employed in ab initio methods are linear combinations of atomic basis functions and since analytic expressions are known for the Fourier transforms of such basis functions,^49^ the transformation of the total molecular electronic wavefunction from position to momentum space is computationally straightforward.^50^

The physical and chemical properties of atoms and molecules strongly depend on the morphology of the density distribution that characterizes the allowed quantum-mechanical states in which the system can be found. The ground-state density, ρ(*r⃗*), is an observable that can be obtained experimentally or calculated using ab initio, semiempirical, or density functional theory methods.^51^

Information theory provides different measures that are able to describe the morphology of the density: the Shannon entropy (localizability), Fisher information (order), disequilibrium (uniformity), and the dyadic product of two single-facet information measures allowing to encompass the complexity of the probability distribution.^32,52−57^

The Shannon entropy, *S*, of a probability density is given by the logarithmic functional^58^
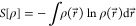
2where ρ(*r⃗*) is the probability density, normalized to unity, in position space that describes the state of a quantum system. This quantity quantifies the total electronic spread in the molecular configuration space; so, it measures the lack of structure of the electron density. Shannon entropy constitutes a measure of the delocalization, i.e., it becomes maximal when the knowledge of ρ(*r⃗*) is minimal. In the case of one-electron atomic systems, the localization of the electron’s position results in an increase of the kinetic energy, and conversely.

The disequilibrium, self-similarity,^59^ or information energy,^60^*D*, quantifies the departure from uniformity of the probability density (equiprobability). In position space, the disequilibrium is defined as^60^
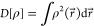
3

These quantities are called global measures as they quantify the total extent of the probability density, scarcely perceiving the fluctuations of the density. In contrast to these two measures, the Fisher information, *I*, has a local character because it is very sensitive to the strong changes in the distribution over a small-sized region. This quantity is given by the following gradient-density functional^31,39^
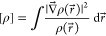
4

Fisher information quantifies the gradient content of the electron distribution, measuring the spatial pointwise concentration of the electronic probability cloud. It can provide a quantitative estimation of the fluctuations of the probability density, thus revealing its irregularities. In addition, this quantity can be interpreted as a measure of the departure of the probability density from disorder, according to the localized/delocalized features of the distributions.

It is interesting to have at our disposal different measures capable of quantifying the complexity of the physical systems beyond the properties of the entropic measures described above. The way to define complexity is not unique: there exits various candidates that can be used as complexity measures, and its quantitative characterization has been an important subject of research during the past decades, and it has received considerable attention. Depending on the type of system or process studied, the level of description, and the scale of interactions between elementary particles, atoms, molecules, biological systems, etc., it may be useful to use one definition or another.

Uncertainty or randomness, clustering, pattern, order, localization, or organization are fundamental concepts for characterizing complexity of physical systems or processes. In this work, we focus our attention in those given in terms of a product of two factors measuring, respectively, order/disorder, localization/delocalization, and randomness or uncertainty of the system under study. In particular, we consider López–Ruiz–Mancini–Calbet (LMC) shape complexity and the Fisher–Shannon (FS) complexities. These two quantities have minimum values for the two extreme probability distributions (perfect order and maximum disorder) and satisfy the desirable properties of dimensionless invariance under scaling transformation, translation, and replication.^61^

The López–Ruíz–Mancini–Calbet (*LMC*) complexity measure,^62,63^*C_LMC_*, is defined by the product of the Shannon exponential entropy *e*^*S*^ and the disequilibrium *D*, both entropic measures of global character
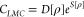
5which jointly describes the average height (uniformity) and the extent of the density (delocalization). This quantity is bounded from below by one, *C_LMC_* ≥ 1, for any three-dimensional probability density.

The Fisher–Shannon (*FS*) complexity,^64,65^*C_FS_*, is also defined by the product of two single entropic quantities: the Fisher information measure *I* (measure with a local character) and the Shannon entropy *S* (measure with a global character), appropriately modified to preserve the common complexity properties
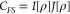
6where  is the Shannon power entropy.

This quantity measures the combined balance of delocalization and order features, i.e., the total spreading and narrowness of the electron density. The Fisher–Shannon complexity measures the gradient content of ρ(*r⃗*) together with its total extent in the support region. This quantity has been employed as a measure of atomic correlation^64^ and also defined as a statistical complexity measure.^66,67^ Moreover, for any three-dimensional probability density, this measure satisfies the inequality *C*_FS_ ≥ 3.

Apart from the explicit dependence on the Shannon entropy that serves to measure the uncertainty (localizability) of the distribution, the Fisher–Shannon complexity has replaced the disequilibrium, present in the LMC complexity, by the Fisher information. This local factor allows us to quantify the departure of the probability density from disorder of a given system through the gradient of the distribution.

As we mentioned above, all information-theoretic quantities are given in atomic units; however, in other units, ρ(*r*) must be explicitly written as (*a*_0_^3^ρ(*r*))/(*Ne*) with the volume element *d*^3^*r*/*a*_0_^3^. For this reason, the information measures are nondimensional. It is worthy to mention that throughout the present study, we have employed electron densities normalized to unity. This choice is convenient in order to employ probability distributions and also to scale densities to the system size through the shape function^53^ (σ(**r**) = ρ(**r**)/*N*).

Note that all measures presented above are defined in the position-space electron density ρ(*r⃗*). However, they can be straightforwardly defined in momentum space through the corresponding momentum density γ(*p⃗*).

## Results and Discussion

The electronic structure calculations were carried out with the G03 and G09 suite of programs^68^ to obtain the wavefunctions and the corresponding densities in conjugated spaces. Necessary geometrical parameters of the TS for the study of the abstraction hydrogenic reaction were employed from the reported data.^69^ The IRC path of the reaction was calculated in the forward/reverse directions of the TS at the UMP2 level of theory that resulted in 35 IRC points evenly distributed in every direction. In the following step, a high level of theory (QCISD(T) method) was employed in a basis set of diffuse and polarized orbitals (6-311++G**) for the calculation of the wavefunction needed for the densities in position and momentum spaces, along with the physicochemical properties of the chemical structures extracted from the IRC. In addition, the *IT* descriptors were obtained by employing software developed in our laboratory along with 3*D* numerical integration routines^70^ and the DGRID suite of programs.^50^ Atomic units are employed throughout the study.

The simplest radical abstraction reaction is the focus of our study: The reaction *H*_*a*_^•^ + *H*_2_ ⇋ *H*_2_ + *H*_*b*_^•^, which involves a reactive intermediate: atomic hydrogen (free radical). The course of the reaction evolves in two steps, *S*_*N*_1 typically. The first stage of the reaction involves the homolysis of the hydrogen molecule to form a new radical (atomic hydrogen). In the second step, the new radical just formed is recombined with another radical species. Such homolytic cleavage occurs when bonding is not polar and there is no other species of electrophilic/nucleophilic type that may cause a heterolytic pattern. When the bond is formed, the product is energetically more stable than the reactants, and therefore bond-breaking requires energy. The above-described mechanistic behavior observed for this type of reaction, in two steps, occurs through an asynchronous behavior. As mentioned above, calculations for this reaction were performed at two different levels: the IRC was obtained at the *UMP2/6-311G* level, and all properties at the IRC were obtained at the QCISD(T)/6-311++G** level of theory. The course of the reaction at the IRC resulted in 72 points uniformly scattered in the forward and reverse directions at the TS. Numerical integrations were performed within a relative error of 1.0 × 10^–5^.^70,71^

In previous studies, the structural features of the distributions in the IRC path of the hydrogenic abstraction reaction have been analyzed for both spaces, position and momentum, focusing on the global features, such as (*delocalization*) through the Shannon entropies,^33^ and (*self-similarity/uniformity*) by employing the disequilibrium measure.^72^ In addition, we also studied the local changes of the distributions for this reaction^34^ by use of the gradient content of the electron distribution that is appropriately described by the Fisher information,^39^ which is a measure of *smoothness/disorder*. The analyses show that each of the *IT* functionals sketched a complementary description of the whole chemical phenomena, due to the global or local characteristics of the particular information measure employed for the analysis. As a result, the hydrogenic abstraction reaction reveals the following chemical regions: R/P (reactant/product complexes), B-B/F (bond-breaking/forming), BCER (bond-cleavage energy reservoirs), and the TS (transition state). In addition, to allow for a complete characterization of the chemical process, it is in our interest to perform a complexity analysis of the reaction to complement the local/global behavior from the single uncertainty measures described above. This is achieved through the *C*_*LMC*_ that describes the joint density features of uniformity and delocalization (eq 5) and also through the *C*_*FS*_ measure, where the delocalization and irregularity features are jointly represented (eq 6). In the following sections, we will recourse to the single and joint uncertainty measures so as to reveal a detailed description of the hydrogenic abstraction reaction.

**Figure 1:**
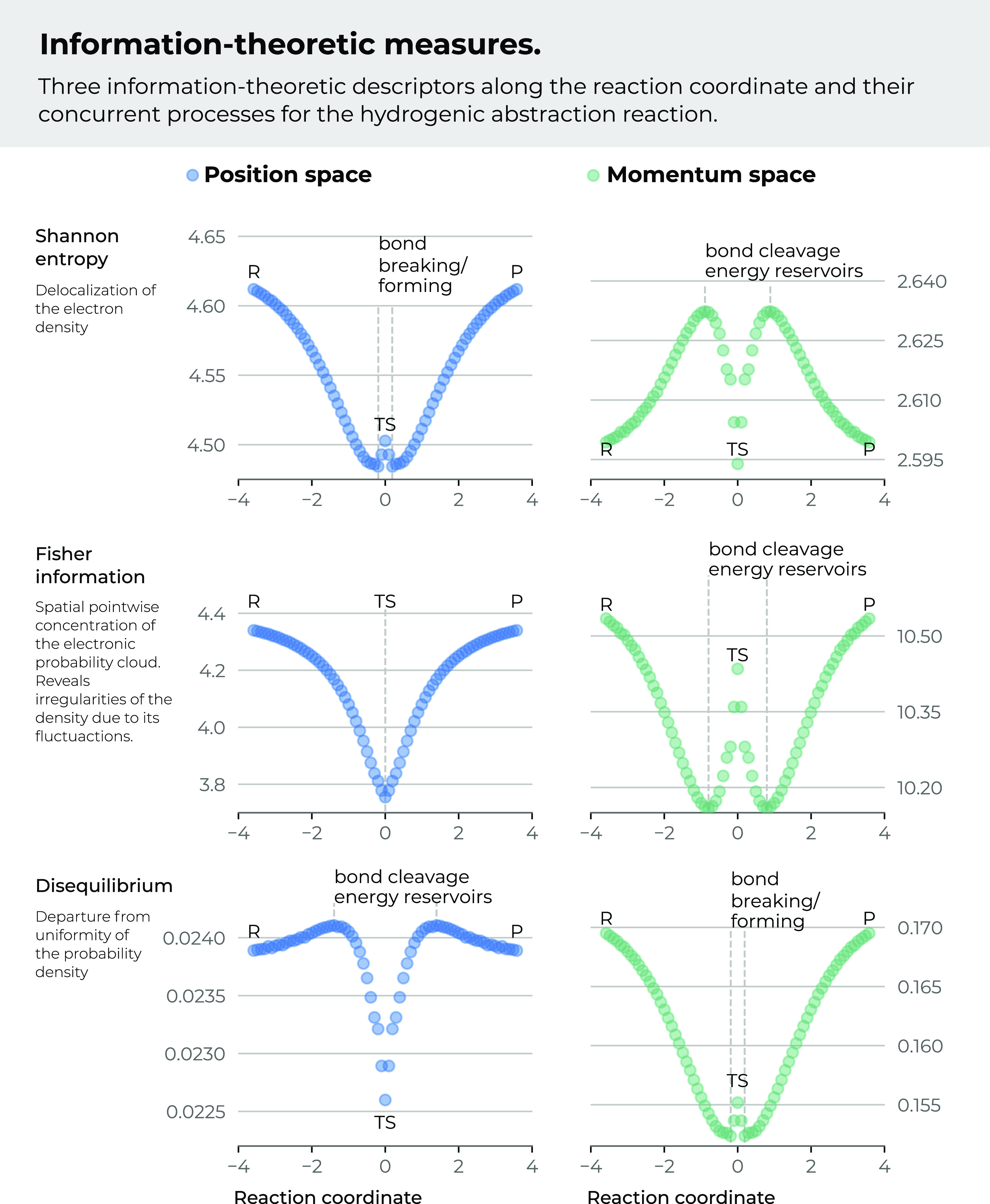
Shannon entropy, Fisher information, and disequilibrium for the IRC of the abstraction reaction in position (left) and momentum (right) spaces.

In Figure 1, we summarize the behavior of the *IT* functionals along the IRC pathway^33,34,72^ in order to guide the 3*D* analysis performed in this study. In this figure, we have depicted the values for the information-theoretic functionals in conjugated spaces to describe the abstraction reaction in terms of *localizability* (Shannon entropies), *uniformity* (disequilibrium measures), and *order* (Fisher information), depending on the magnitude of the *IT* measures.

Figure 1 summarizes the phenomenological behavior of the chemical process through the Shannon entropy:^33^ as the molecular complex approaches the TS, its position-space density becomes localized (minima of entropy in position space *S*_*r*_) at the onset of the bond-breaking region, and such a process would require energy to proceed, which is revealed by the local maximum in the corresponding density in momentum space, i.e., its delocalization is indicative of the local increment of kinetic energy required for bond-breaking at the BCER. Next, the homolysis provokes energetic relaxation of the molecular densities approaching the TS (see Figure 1).

Next, the Fisher information is employed to reveal the local features of the distributions at the IRC, *I* (departure from disorder).^34,72^ In terms of this quantity, the phenomenological behavior of the chemical course of the reaction can be summarized as follows: Fisher information (position space) holds maxima at the R/P regions and global minimum at the TS. This is chemically interpreted as follows: as the reactant molecular complex approaches the TS, the distributions in position space become *disordered*; hence, the structural changes of the distributions diminish as the reaction evolves. Moreover, at the R/P regions, Fisher shows larger changes than the TS, showing the smoothest density profile as compared with the rest along the IRC; this might be associated with the spin-coupling of the hydrogenic radical species. In contrast, the gradient of the density increases toward the BCER at the onset of the bond-cleavage region. On the other hand, in momentum space, we have observed^34,72^ that Fisher information *I*_*p*_ reveals highly *ordered* structures at the R/P regions (lower kinetic energies) as well as the TS, showing the largest Fisher values, which according to Figure 1, correspond to the most *localized* distributions in position space, whereas the BCER were associated with the most *disordered* structures (higher kinetic energies) that are obviously linked with the most *delocalized* momentum densities.

On the side of the disequilibrium functional in position space, *D*_*r*_, we observed in a previous work^72^ that at the R/P regions, *D*_*r*_ shows lower values than at the BCER regions, whereas it holds a global minimum at the TS region, i.e., the position-space distributions are the least *uniform* at the BCER, whereas the most *uniform* distribution is observed at the TS. Chemically, the densities at the R/P region exert larger changes so as to reach maxima at the BCER. These regions are also associated with the most *delocalized* densities in momentum space according to the Shannon entropy *S*_*p*_, hence corresponding to the most energetic structures at the IRC. The whole process proceeds in two steps: at the end of the bond-cleavage process, the TS shows the most *uniform* distribution during the first step of the reaction.

Chemically, the R/P and the TS regions in momentum space exert larger energetic changes as compared to the BCER regions by augmenting the kinetic energy necessary for bond-breaking at the BCER, and this is achieved by deforming the ionic complex when passing from ordered momentum densities at the R-region in the forward direction of the reaction, up to the disordered one at the BCER reservoir, and then releasing that energy when bond cleavage occurs so as to form the TS complex that is characterized by a significantly more ordered distribution in momentum space (Fisher locally maximal) in the first step of the reaction. In the second step, the new bond is formed beyond the TS. Indeed, at the onset of this region, the process gets reverted toward the P-region by releasing the accumulated energy at the TS when bond cleavage is completed and then a more *disordered* structure with a larger kinetic energy is reached at the BCER, the reaction continues in this second step so as to employ the accumulated energy for the bond-forming process at the BCER to release it again so as to reach the product complex (P-region). The complete view of the two-step mechanism of the abstraction reaction may be assessed by the global features of the disequilibrium measure that shows that the R/P possesses the least *uniform* momentum-space densities among the rest at the IRC, whereas the B-B/F regions hold the most *uniform* ones. Note that minima of *I* indicate the BCER reservoirs, whereas minima of *D* indicate the B-B/F regions. The TS structure is characterized by a local maximum with a less *uniform* momentum density. Furthermore, at the onset of the TS region in both directions, a very distinctive feature of the functional attributes was observed from both measures; interestingly, the abrupt structural energetic change observed at the TS from the Fisher information is much more pronounced than the one required for the bond-breaking process, the region where the B-B/F occurs according to the D measure, indicating both that the concurrent phenomenon of spin-coupling is the most important one, driving the course of the reaction and the reason why this chemical process occurs in two steps. Therefore, it was concluded that these *IT* functionals are complementary, energetically (*I*) and structurally (*D*). For a more detailed discussion see ref (72).^72^

**Figure 2:**
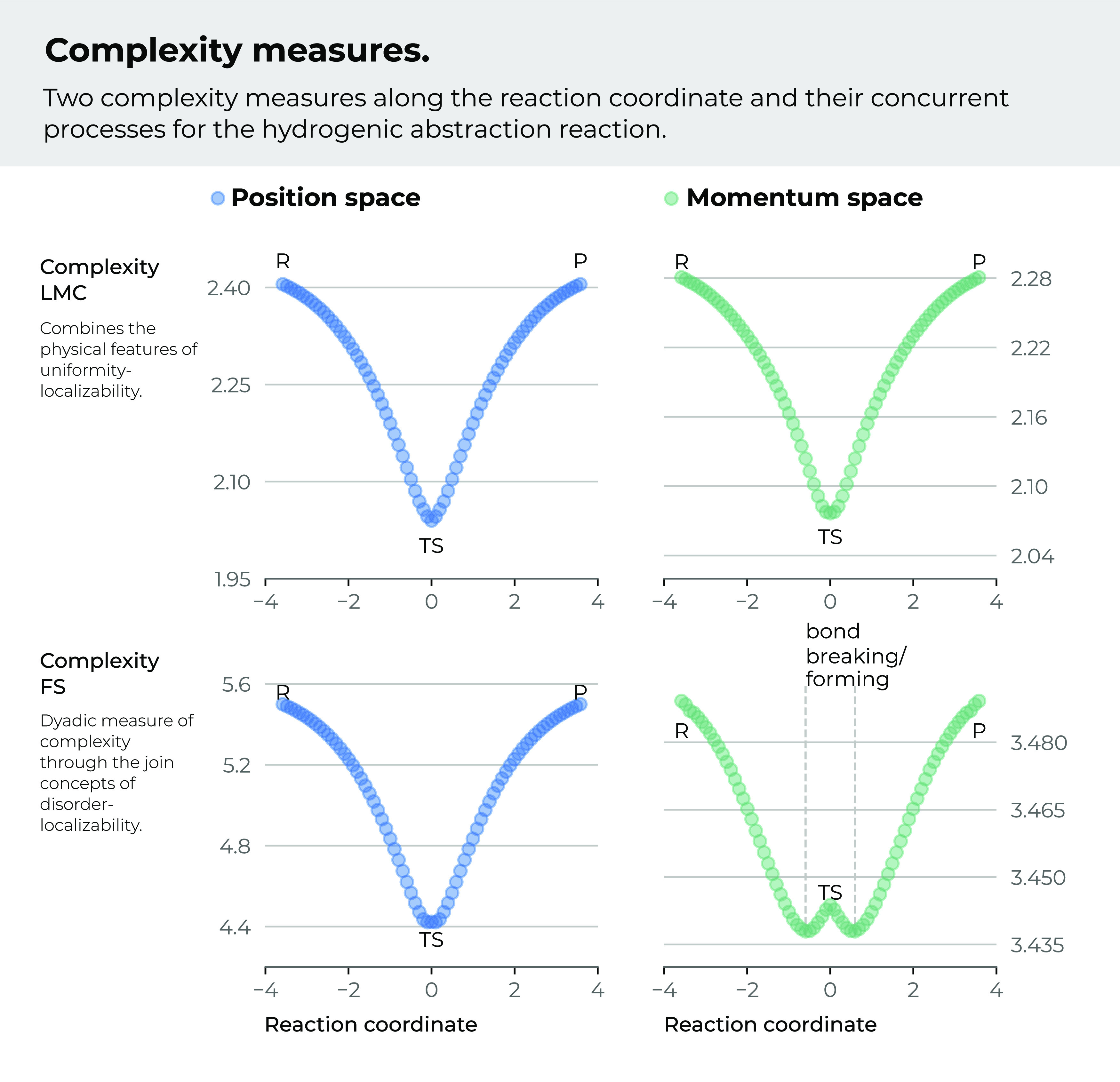
*LMC* complexity, *C*_*LMC*_, and Fisher–Shannon complexity, *C*_*FS*_, measures for the IRC of the abstraction reaction in position (left) and momentum (right) spaces.

In a previous study, we have analyzed the dyadic measures of *C*_*LMC*_, which combine the physical features of *uniformity-localizability* and *C*_*FS*_ that does it through the joint concepts of *disorder-localizability*.^72^ In Figure 2, the values for these complexity measures are shown in position and momentum spaces, respectively. We observe from the figure that both measures represent similar situations in position space. It is shown that the R/P regions hold maximum complexity, and as the reaction evolves, both complexities diminish up to the TS, which holds the minimum complexity value at the IRC. We have noted that both measures neither detect the BCER nor the B-B/F regions. In momentum space, we can note that the *C*_*LMC*_ behaves similar to that in position space; that is, it shows maxima for the R/P regions and its minimal for the TS, again failing to detect the BCER and the B-B/F regions. In contrast, *C*_*FS*_ behaves differently, showing maximum values at the R/P regions and minima at the B-B/F regions, with a local maximum at the TS. It is worth noting that neither of the two separate measures of *I*_*p*_ and *S*_*p*_ were able to detect the B-B/F regions (see Figure 1), whereas the joint measure can do it.

### Hypersurface of Information-Theoretic Functionals and Complexity Measures

At the light of the discussions above, it would be of interest to analyze the course of the abstraction reaction within the framework of a 3*D* analysis in conjugated spaces so as to examine the chemical neighborhood of the *IT* functionals and complexity measures in 3D space. It is important to mention that all properties were calculated in a grid of the internal coordinates of the three hydrogenic complex *H*_*a*_ ··· *H*_*b*_ ··· *H*_*c*_ from 0.5*a.u*. ≤ *R*_12_ ≤3.35a*.u*. vs. 0.5*a.u*. ≤ *R*_13_ ≤ 3.35*a.u*. in steps of 0.05*a.u*. As mentioned above, the electron densities in conjugated spaces necessary to construct the hypersurface of the *IT* functionals were calculated at the QCISD(T) level of theory in a 6-311++G** basis set by use of standard computational chemistry programs^50,68,70^ and software developed in our laboratory.

**Figure 3:**
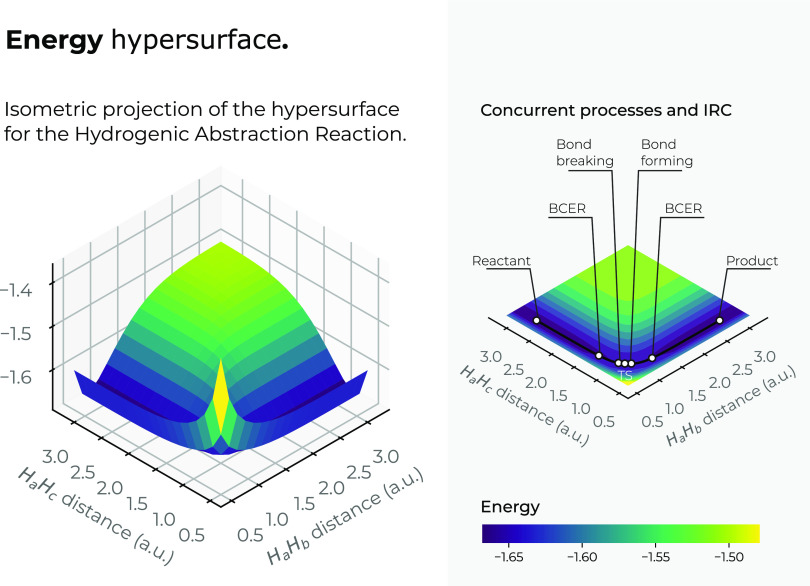
Total energy hypersurface (left) and top view of its hypersurface in the *X*–*Y* plane (right), of the hydrogenic abstraction reaction in the grid of the internal coordinates, *R*_12_ and *R*_13_, of the three hydrogenic complex *H*_*a*_ ··· *H*_*b*_ ··· *H*_*c*_. Color codes indicate lower to higher energy values running from bluish to yellowish ones, respectively.

We have found useful to draw the total energy surface in 3*D* as a function of the internal coordinates *R*_12_ and *R*_13_, which is depicted in Figure 3. A top view of the 3*D* energy surface in the *X*–*Y* plane is also outlined in the figure, indicating the IRC pathway of the reaction; details of the R/P, B-B/F, BCER, and TS regions are also shown. Color codes indicate lower to higher energy values running from bluish to yellowish ones, respectively. The general observation from Figure 3 is that the IRC pathway runs upstream from the reactants region, in the forward direction of the reaction, up to the TS, signaling the energy maximum at the saddle point of the basin surface and then it runs downstream along the valley to reach the products. It is interesting to note that the IRC represents the minimum energy channel along the steep-sided valley. Noteworthy is that the extrema of the IRC at the regions of interest, R/P, B-B/F, BCER, and TS, cannot be perceived by the energy, except by the R/P and TS regions. The B-B/F and BCER are also depicted though.

#### 3*D* Surfaces in Position Space

**Figure 4:**
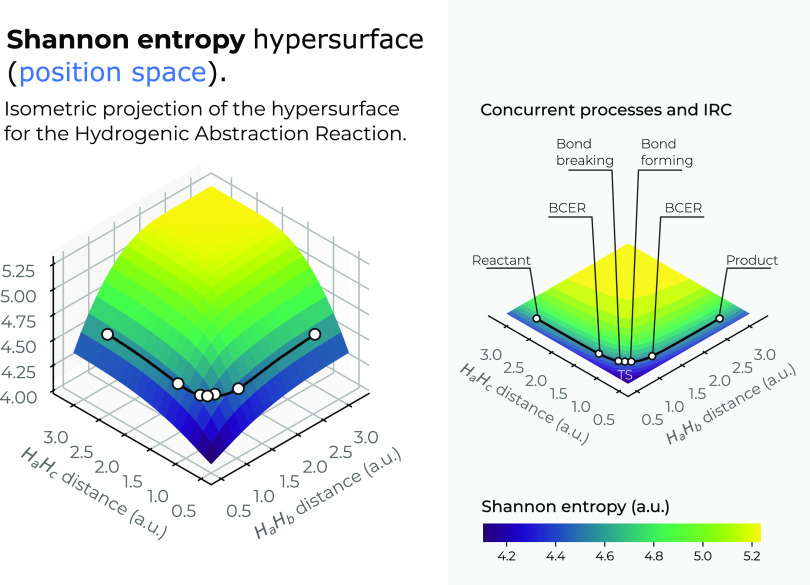
Hypersurface of the Shannon entropy in position space (left) and top view of its hypersurface in the *X*–*Y* plane (right) for the hydrogenic abstraction reaction in the grid of the internal coordinates, *R*_12_ and *R*_13_, of the three hydrogenic complex *H*_*a*_ ··· *H*_*b*_ ··· *H*_*c*_. The IRC path is indicated in a black solid line along with the important regions of the reaction R/P, B-B/F, BCER, and TS. Color codes indicate lower to higher entropy values running from bluish to yellowish ones, respectively.

The position-space Shannon entropy surface in 3*D* is shown in Figure 4 as a function of the internal coordinates *R*_12_ and *R*_13_. We also plot a top view of the *X*–*Y* plane of the 3*D* entropy surface, indicating the IRC pathway of the reaction; the details of the R/P, B-B/F, BCER, and TS regions are also shown in a black line. Color codes indicate lower to higher entropy values running from bluish to yellowish ones, respectively.

We may observe from Figure 4 that the IRC pathway runs at the steep side of a deep slope falling downstream where the entropy holds local minima, a physical situation where the triatomic molecular complex shows a highly localized density (see Figure 1) in preparation for bond rupture. Next, the homolysis provokes energy/density relaxation of the molecular complex at the TS where the entropy reaches its local maxima by delocalizing the position-space density. Upstream, at the steep side of the slope, the chemical situation gets inverted so as to reach local minima at the P-region where the position density gets localized. Note from Figure 4 that the chemical regions of interest, the R/P, B-B/F, BCER, and TS, along the IRC pathway, run at the folded side of a saddle-like surface. This observation possesses an interesting possibility of designing multiple entropy-driven reactions, parallel to the entropy-driven IRC pathway, downstream of the steep side of the saddle, for excited molecular states of the same reaction though. This observation deserves to be further investigated to assess its practical utility. It is noteworthy that all of the extrema of the IRC for the Shannon entropy are shown at the steep side of the valley (see Figure 1 for a detailed description), at the Shannon’s saddle surface, which is in contrast with the 3*D* energy surface, which runs along the bed of the channel.

**Figure 5:**
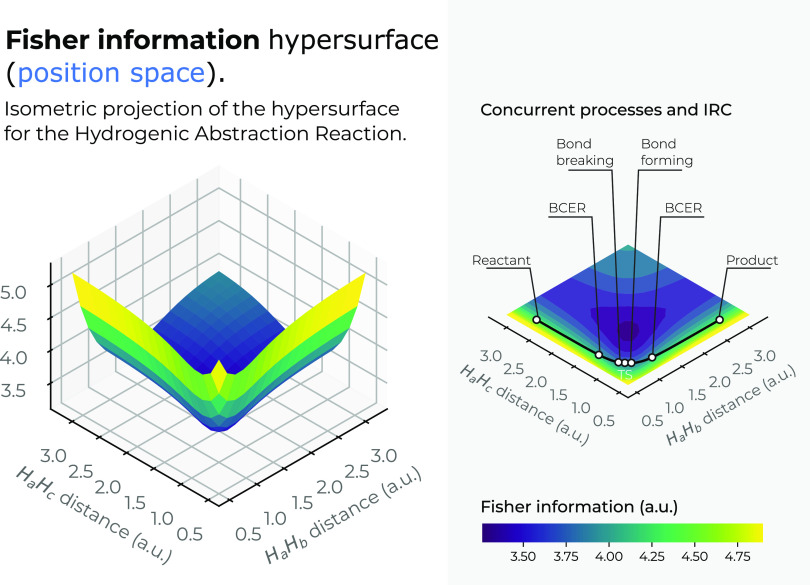
Hypersurface of the Fisher information in position space (left) and top view of its hypersurface in the *X*–*Y* plane (right), for the hydrogenic abstraction reaction in the grid of the internal coordinates, *R*_12_ and *R*_13_, of the three hydrogenic complex *H*_*a*_ ··· *H*_*b*_ ··· *H*_*c*_. The IRC path is indicated in a black solid line along with the important regions of the reaction R/P, B-B/F, BCER and TS. Color codes indicate lower to higher Fisher values running from bluish to yellowish ones, respectively.

Fisher information surface in 3*D* in position space is depicted in Figure 5 as a function of the internal coordinates *R*_12_ and *R*_13_. A top view of the *X*–*Y* plane of the Fisher’s 3*D* surface is also shown, indicating the IRC pathway of the reaction; the details of the R/P, B-B/F, BCER, and TS regions are also shown in a black solid line. Color codes indicate lower to higher Fisher values running from bluish to yellowish ones, respectively. We may observe from Figure 5 that the IRC pathway runs at the steep side of a deep narrow Fisher well falling downstream at the bottom where Fisher holds its lowest values (highly ordered structures) at the expense of stretching the ionic molecular complex. It would be of interest to further study the nature of this molecular complex that seems to hold some kind of structural stability, although it certainly must be some sort of stable excited state. Let us briefly mention that there exists the protonated molecule of hydrogen, a triatomic cationic species that is very abundant in stellar gases.^73^ Naturally, when the molecular complex dissociates, Fisher indicates a chemical situation of higher disorder. Note from Figure 5 that the chemical regions of interest, the R/P, B-B/F, BCER, and TS, along the IRC pathway, run at the internal-folded side of an inverted saddle as Shannon does; although the structures of the basins are very different, a steepest descending surface is observed for the entropy (see Figure 4), whereas the Fisher surface is located around the well. Interestingly, all of the extrema of the IRC for the Fisher functional in 3*D* are shown at the steep side of the valley, halfway of the reverse side of the Fisher’s saddle, which is in contrast with the 3*D* energy surface, which runs along the bed of the channel.

**Figure 6:**
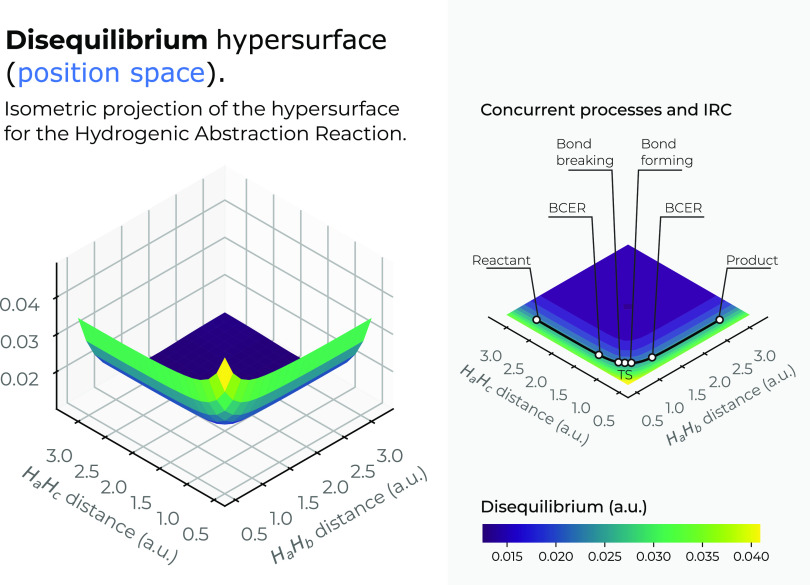
Hypersurface of the disequilibrium functional in position space (left) and top view of its hypersurface in the *X*–*Y* plane (right) for the hydrogenic abstraction reaction in the grid of the internal coordinates, *R*_12_ and *R*_13_, of the three hydrogenic complex *H*_*a*_ ··· *H*_*b*_ ··· *H*_*c*_ (0.5*a.u*. ≤ *R*_1*j*_ ≤ 3.35*a.u*., *j* = 2, 3 in steps of 0.05*a.u*.). Color codes indicate lower to higher disequilibrium values running from yellowish to bluish ones, respectively.

In Figure 6, we have depicted the disequilibrium surface in 3*D* in position space as a function of the internal coordinates *R*_12_ and *R*_13_. In this figure, we plot a top view of the *X*–*Y* plane of the disequilibrium’s 3*D* surface, indicating the IRC pathway of the reaction; details of the R/P, B-B/F, BCER, and TS regions are also shown in a black solid line. Color codes indicate lower to higher disequilibrium values running from bluish to yellowish ones, respectively. We note from Figure 6 a similar pattern to that of the Fisher information (see Figure 5), i.e., the structural features of the 3*D* surface for both *D* and *I* are practically the same. Figure 6 shows that the IRC pathway runs at the steep side of a deep wider slope falling downstream at the bottom where disequilibrium holds its lowest values, corresponding to highly *uniform* densities at the expense of stretching the ionic molecular complex through their dissociation process. Upstream, at the top of the valley, the disequilibrium reaches its larger values (least uniform distributions, highly distorted) when the ionic molecular complex tends to collapse. Note from Figure 6 that the chemical regions of interest, the R/P, B-B/F, BCER, and TS, along the IRC pathway, run at the internal-folded side of an inverted saddle.

**Figure 7:**
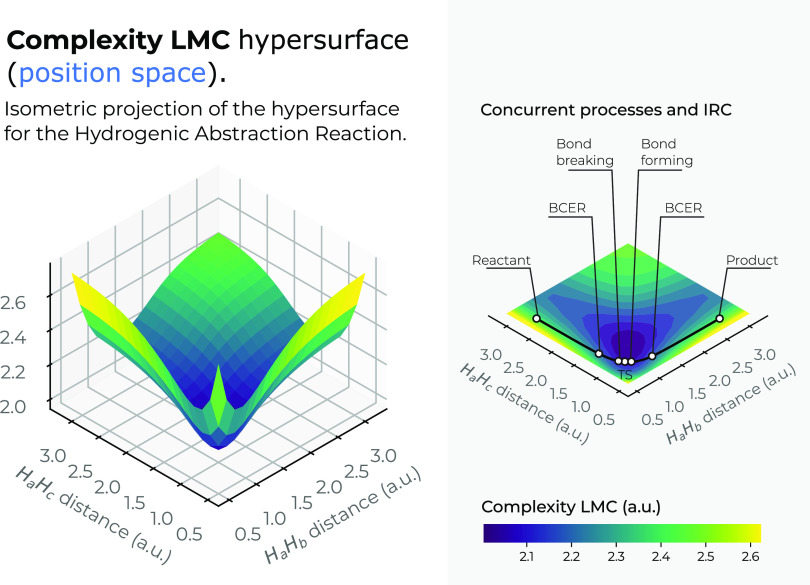
Hypersurface of the *LMC* complexity measure in position space (left) and top view of its 3*D* surface in the *X*–*Y* plane (right) for the hydrogenic abstraction reaction in the grid of the internal coordinates, *R*_12_ and *R*_13_, of the three hydrogenic complex *H*_*a*_ ··· *H*_*b*_ ··· *H*_*c*_. Color codes indicate lower to higher *LMC*-complexity values running from yellowish to bluish ones, respectively.

**Figure 8:**
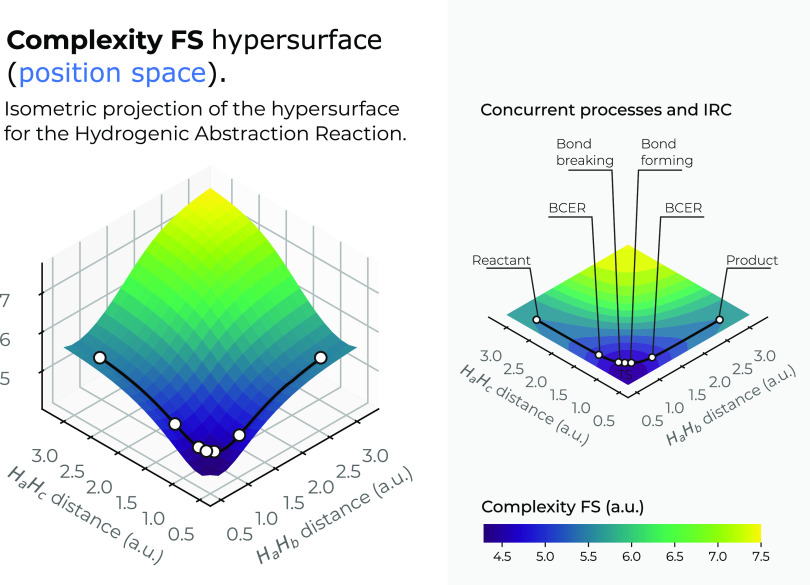
Hypersurface of the Fisher–Shannon complexity measure in position space (left) and top view of its 3*D* surface in the *X*–*Y* plane (right) for the hydrogenic abstraction reaction in the grid of the internal coordinates, *R*_12_ and *R*_13_, of the three hydrogenic complex *H*_*a*_ ··· *H*_*b*_ ··· *H*_*c*_. Color codes indicate lower to higher Fisher–Shannon complexity values running from yellowish to bluish ones, respectively.

In Figure 7, we have drawn the *LMC* complexity surface in 3*D* in position space as a function of the internal coordinates *R*_12_ and *R*_13_. A top view in the *X*–*Y* plane of the hypersurface of *C*_*LMC*_ complexity measure is also depicted in the figure. In Figure 8, a similar plot is depicted for the *C*_*FS*_ complexity measure, indicating the IRC pathway of the reaction in both figures along with the chemical interesting zones: R/P, B-B/F, BCER, and TS. Color codes indicating lower to higher values for the complexities from bluish to yellowish ones, respectively.

Figure 7 shows a deep narrow well that encloses all of the important regions of the reaction, where the concurrent phenomena occur, i.e., the B-B/F, BCER and the TS regions, indicating that chemically important changes occur at lower regions of *LMC* complexity (greenish complexity regions in Figure 7). It would be interesting to further investigate the chemical consequences of the existence of this low complexity well that might indicate some kind of boundary delimiting structurally stable molecules. It is interesting to note that Fisher’s hypersurface in Figure 5 indicates a similar region, which is a different kind of functional to the global ones employed to represent the *C*_*LMC*_. In other words, this low-complexity boundary is detected by local and global IT functionals. Hence, it seems the deep narrow well represents indeed a chemically interesting boundary. On the other hand, the 3*D* surface of *C*_*LMC*_ shows that higher *LMC*-complexity regions are found at the dissociated complex species, i.e., (*H*_*a*_ ··· *H*_*b*_ ··· *H*_*c*_)^·^ → *H*_2_ + *H*_*c*_^·^ at very large *R*_*bc*_, (*H*_*a*_ ··· *H*_*b*_ ··· *H*_*c*_)^·^ → *H*_*a*_^·^ + *H*_2_ at very large *R*_*ab*_, and (*H*_*a*_ ··· *H*_*b*_ ··· *H*_*c*_)^·^ → (*H*_*a*_ + *H*_*b*_ + *H*_*c*_)^·^ at very large *R*_*aBocbc*_. We conclude that dissociated species are informationally complex.

Figure 8 shows a deep narrow slope at small distances of the internal coordinates *R*_12_ and *R*_13_, indicating a low Fisher–Shannon complexity, *C*_*FS*_, region that interestingly also encloses the chemically interesting regions at the concurrent phenomena zone (B-B/F, BCER, and TS) similar to the *LMC* complexity (Figure 7). Further, higher Fisher–Shannon complexity regions are found at the dissociation zone of this complex radical molecule: (*H*_*a*_ ··· *H*_*b*_ ··· *H*_*c*_)^·^ → (*H*_*a*_ + *H*_*b*_ + *H*_*c*_)^·^ at very large *R*_*abc*_, in contrast with the *LMC* complexity that shows higher values for all kinds of dissociated species (see above).

Boundaries of low-complexity regions for both measures, *C*_*LMC*_ and *C*_*FS*_, enclose complex radical molecules with lower complexity values than the TS, as may be observed from Figures 7 and 8, which is chemically interesting, and we think that deserves further investigation. Moreover, another interesting observation from these figures allows us to note that the hypersurface for both complexities in position space folds around the TS region, revealing an attractor-like spatial zone.

#### 3*D* Surfaces in Momentum Space

**Figure 9:**
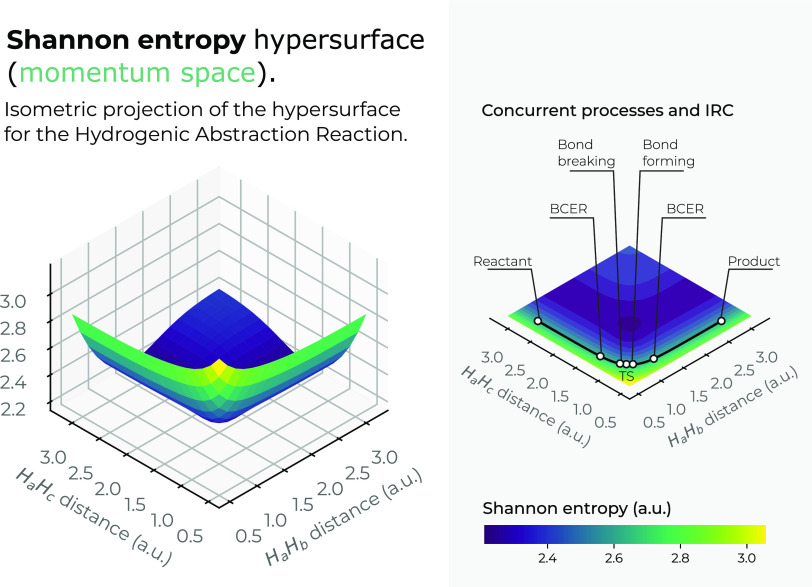
Hypersurface of the Shannon entropy in position space in momentum space (left), and top view of the Shannon’s 3*D* surface in the *X*–*Y* plane (right) for the hydrogenic abstraction reaction in the grid of the internal coordinates, *R*_12_ and *R*_13_, of the three hydrogenic complex *H*_*a*_ ··· *H*_*b*_ ··· *H*_*c*_. Color codes indicate lower to higher Shannon entropy values running from yellowish to bluish ones, respectively.

**Figure 10:**
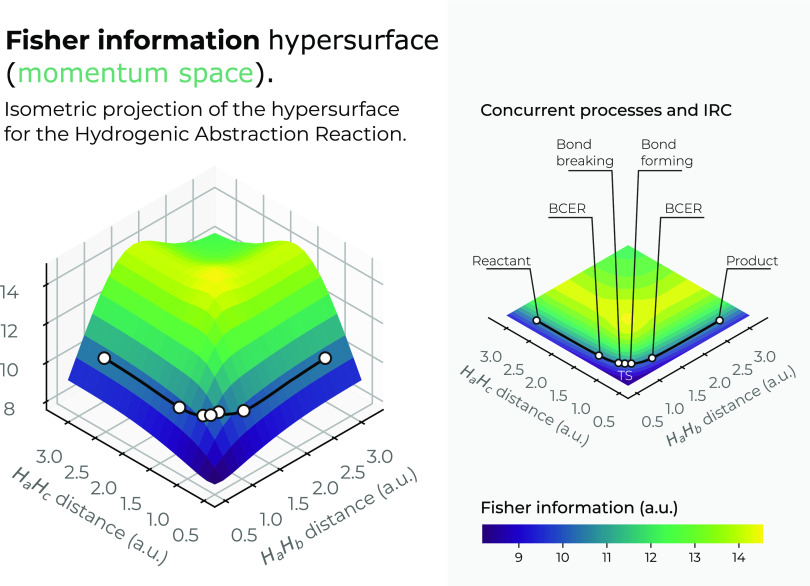
Hypersurface of the Fisher information in position space in momentum space (left), and top view of the Fisher’s 3*D* surface in the *X*–*Y* plane (right) for the hydrogenic abstraction reaction in the grid of the internal coordinates, *R*_12_ and *R*_13_, of the three hydrogenic complex *H*_*a*_ ··· *H*_*b*_ ··· *H*_*c*_. Color codes indicate lower to higher Fisher information values running from yellowish to bluish ones, respectively.

**Figure 11:**
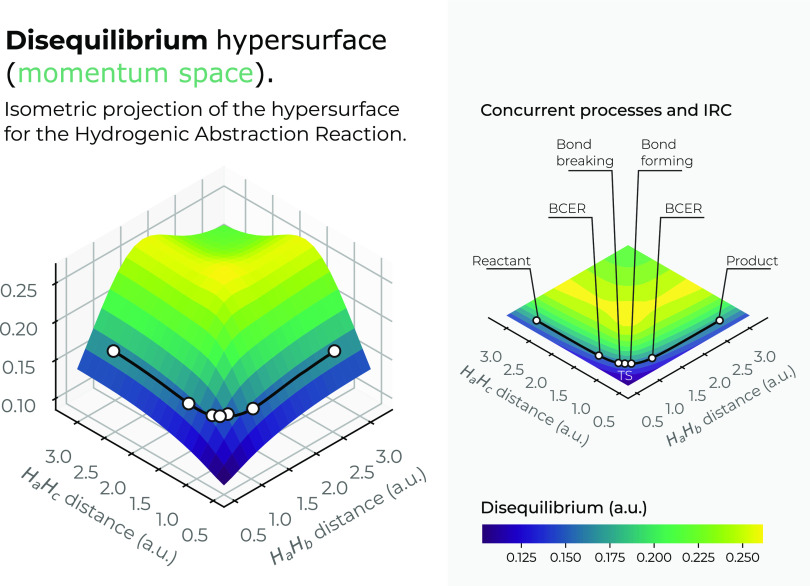
Hypersurface of disequilibrium in momentum space *D*_*m*_ for the hydrogenic abstraction reaction in the grid of the internal coordinates, *R*_12_ and *R*_13_, of the three hydrogenic complex *H*_*a*_ ··· *H*_*b*_ ··· *H*_*c*_ (0.5*a.u*. ≤ *R*_1*j*_ ≤ 3.35*a.u*., *j* = 2, 3 in steps of 0.05*a.u*.). Color codes indicate lower to higher disequilibrium values running from yellowish to bluish ones, respectively.

The momentum-space surfaces in 3*D* for the IT functionals *S*_*m*_, *I*_*m*_, and *D*_*m*_ are drawn in Figures 9–11. It is apparent that a very different shape is shown in momentum space as compared to position space in 3*D*. For instance, the Shannon entropy in momentum space *S*_*m*_, depicted in Figure 9, shows a deep narrow valley falling downstream up to a low-entropy basin, forming the reverse side of the horse saddle. Let us recall that entropy in position space does not hold such a channel. Although the IRC pathway is not indicated in the figure, it is easy to locate at *R*_12_ = *R*_13_ ≈ 1.75*a.u*. (yellowish-greenish surface regions in the figure). It is worth mentioning that the low-entropy channel at the bottom of the entropy hypersurface is not expected and deserves further investigation. Highly localized momentum densities should indicate low kinetic energy structures, i.e., stable complex molecules. On the other hand, the local behavior of the momentum density is sketched in Figure 10 through the *I*_*m*_ functional that shows a deep narrow slope in the upside of the saddle, which is in contrast with Fisher in position space, which shows the opposite behavior. From Figure 10, we can observe low values of *I*_*m*_ at the IRC pathway, i.e., which correspond to low-ordered momentum densities thus characterizing the IRC and the important chemical regions of the reaction. Of course, a more detailed analysis of the course of the reaction reveals more information as we discussed in previous sections; however, at this stage of the analysis, we would like to assess the region wherein the IRC is located in the whole chemical space. Furthermore, in connection with the *D* – *m* measure, it is worth noting the similarity among both hypersurfaces for *I* and *D*, shown in Figures 10 and 11, revealing a deep narrow slope in the upside of the saddle. In Figure 11, we observe that low values of disequilibrium in momentum space characterize the IRC pathway, i.e., highly uniform momentum densities. Again, a thorough analysis of the structure of the IRC pathway for this property has been discussed above.

**Figure 12:**
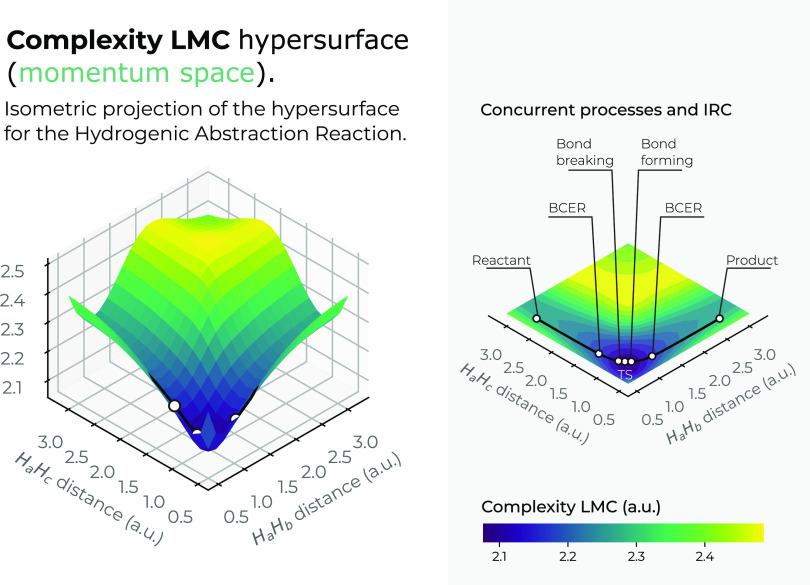
Hypersurface of the *LMC* complexity measure in momentum space in position space (left), and top view of the complexity’s 3*D* surface in the *X*–*Y* plane (right) for the hydrogenic abstraction reaction in the grid of the internal coordinates, *R*_12_ and *R*_13_, of the three hydrogenic complex *H*_*a*_ ··· *H*_*b*_ ··· *H*_*c*_. Color codes indicate lower to higher *LMC* complexity values running from yellowish to bluish ones, respectively.

**Figure 13:**
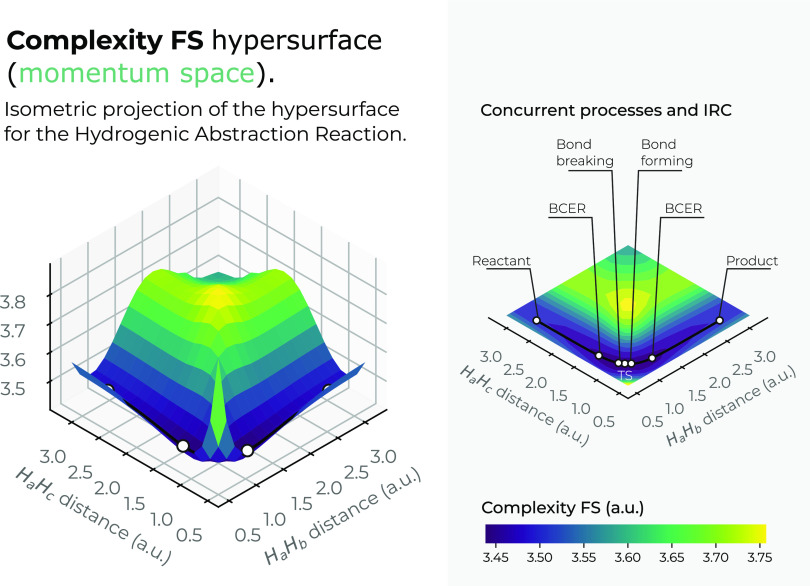
Hypersurface of the Fisher–Shannon complexity measure in momentum space in position space (left), and top view of the complexity’s 3*D* surface in the *X*–*Y* plane (right) for the hydrogenic abstraction reaction in the grid of the internal coordinates, *R*_12_ and *R*_13_, of the three hydrogenic complex *H*_*a*_ ··· *H*_*b*_ ··· *H*_*c*_. Color codes indicate lower to higher Fisher–Shannon complexity values running from yellowish to bluish ones, respectively.

The momentum-space surfaces in 3*D* for the complexity measures *C*_*LMC*_ and *C*_*FS*_ are plotted in Figures 12 and 13. Interestingly, both complexity measures show low-complexity regions in the chemically interesting regions of the reaction at the IRC pathway, *R*_12_ = *R*_13_ ≈ 1.75*a.u*. (greenish surface regions in both figures), where the concurrent phenomena occur. Hence, it seems that an interesting conclusion of this study reveals that chemical reactions occur within the boundaries of low-complexity IT-space regions. Also, it is noteworthy that Fisher–Shannon complexity in momentum space shows much more structure than its corresponding one in position space, the former showing a low-complexity channel along the upside of the saddle and the latter a deep narrow slope, which drives the reaction path toward the transition region where the concurrent phenomena occur and to the transition state.

Interestingly, from Figures 12 and 13, we observe the same behavior as in position space, i.e., the hypersurface for both complexities in momentum space fold around the TS region, revealing an attractor-like momentum zone.

## Conclusions

We have undertaken a study of the course of an elementary chemical reaction from the perspective of information-theoretic (IT) in 3*D* space through the hypersurface of several information functionals such as disequilibrium (*D*), Shannon entropy (*S*), Fisher information (*I*), and the Fisher–Shannon (*FS*) and López–Mancini–Calbet *LMC* shape complexities. The probe for the study is the hydrogenic identity abstraction reaction.

The 3*D* analyses revealed interesting reactivity patterns in the neighborhood of the intrinsic reaction coordinate (IRC) path that allow to interpret the reaction mechanism for this reaction in a novel manner. In addition, the chemically interesting regions that have been characterized through the information functionals and their complexity measures are depicted and analyzed in the framework of the three-dimensional structure of the information-theoretical data of a chemical reaction, that is, the reactant/product (R/P) complexes, the transition state (TS), and the ones that are only revealed through (*IT*) measures such as the bond-cleavage energy region (BCER), the bond-breaking/forming (B-B/F) regions, and the spin-coupling (SC) process. Furthermore, focus has been placed on the diagonal part of the hypersurface of the IT functionals, aside from the IRC path itself, with the purpose of analyzing the dissociation process of the triatomic transition-state complex that has revealed other interesting features of the bond-breaking (B-B) process. It is shown throughout the combined analyses of the 3*D* structure of the (*IT*) functionals that the chemically significant regions occurring at the onset of the TS are completely characterized by information-theoretic aspects of localizability (*S*), uniformity (*D*), and disorder (*I*). Further, novel regions of low complexity seem to indicate new boundaries for chemically stable complex molecules. Finally, the study reveals that the chemical reaction occurs at low-complexity regions, where the concurrent phenomena take place: bond-breaking/forming (B-B/F), bond-cleavage energy reservoirs (BCER), spin-coupling (SC), and transition state (TS) occur. Further, complexity measures in both spaces show folded hypersurfaces around the TS region, revealing attractor-like spatial/momentum zones.

An intriguing proposition has been brought up to our attention in that isotopic substitution by deuterium (D) replacing hydrogen would reflect the same conclusions? At first sight, it would appear as deuteron substitution should not affect the course of the reaction on the grounds that chemistry is about the last shell of electrons in atoms, whereas nuclear differences are in the field of Nuclear Physics. Apparently, no fundamental differences would be expected in the chemical behavior of reactions since D is only a nuclear isotope of H, both with the same electrostatic interaction. However, vibration movement is about nuclear masses (neglecting the electron mass); therefore, we would expect different vibrational energies that produce lower zero-point energies for H – H, H – D, and D – D; consequently, larger energies are required to break the deuterium bonds. Also, depending on the reaction *D*^•^ + *H* – *H* ⇋ *H* – *D* + *H*^•^ or *D*^•^ + *H* – *D* ⇋ *D* – *D* + *H*^•^, we would also expect nonsymmetric energetic profiles and, consequently, different (nonsymmetric) information-theoretic profiles too. The question deserves further research and it is indeed a perspective for future investigation.
